# Structural and Functional Changes in Non-Paraneoplastic Autoimmune Retinopathy

**DOI:** 10.3390/diagnostics13213376

**Published:** 2023-11-03

**Authors:** Amir Akhavanrezayat, Anadi Khatri, Neil Gregory L. Onghanseng, Muhammad Sohail Halim, Christopher Or, Nripun Sredar, Moataz Razeen, Murat Hasanreisoglu, Jonathan Regenold, Zheng Xian Thng, S. Saeed Mohammadi, Tanya Jain, Negin Yavari, Vahid Bazojoo, Ankur Sudhir Gupta, Azadeh Mobasserian, Cigdem Yasar, Ngoc Trong Tuong Than, Gunay Uludag Kirimli, Irmak Karaca, Yong-Un Shin, Woong-Sun Yoo, Hashem Ghoraba, Diana V. Do, Alfredo Dubra, Quan Dong Nguyen

**Affiliations:** 1 Spencer Center for Vision Research, Byers Eye Institute, Stanford University School of Medicine, 2370 Watson Court, Palo Alto, CA 94303, USA; akhavana@stanford.edu (A.A.); anadikc@stanford.edu (A.K.);; 2 Birat Aankha Aspatal, Biratnagar 56613, Nepal; 3 Department of Ophthalmology, Birat Medical College and Teaching Hospital, Kathmandu University, Biratnagar 45200, Nepal; 4 Department of Ophthalmology, Makati Medical Center, Manila 1229, Philippines; 5 Ocular Imaging Research and Reading Center, Sunnyvale, CA 94085, USA; 6 Department of Ophthalmology, Koc University School of Medicine, 34450 Istanbul, Turkey; 7 Koc University Research Center for Translational Medicine, Koc University, 34450 Istanbul, Turkey; 8 National Healthcare Group Eye Institute, Tan Tock Seng Hospital, Singapore 308433, Singapore; 9 Dr. Shroff Charity Eye Hospital, New Delhi 110002, India; 10 Department of Ophthalmology, Duke University, Durham, NC 27705, USA; 11 Department of Ophthalmology, Hanyang University Guri Hospital, Hanyang University College of Medicine, Seoul 04763, Republic of Korea; 12 Department of Ophthalmology, Gyeongsang National University College of Medicine, and Gyeongsang National University Hospital, Jinju 52727, Republic of Korea

**Keywords:** non-paraneoplastic autoimmune retinopathy, AIR, functional changes, structural changes, retina imaging, AOSLO, adaptive optics, Goldmann visual field, GVF, microperimetry

## Abstract

Background: To describe longitudinal changes in patients with non-paraneoplastic autoimmune retinopathy (npAIR) by utilizing different diagnostic modalities/tests. Methods: The index study is a retrospective longitudinal review of sixteen eyes of eight patients from a tertiary care eye hospital diagnosed with npAIR. Multiple diagnostic modalities such as wide-angle fundus photography (WAFP), WA fundus autofluorescence (WAFAF), spectral-domain optical coherence tomography (SD-OCT), Goldmann visual field (GVF) perimetry, microperimetry (MP), electrophysiologic testing, and adaptive optics scanning laser ophthalmoscopy (AOSLO) were reviewed and analyzed. Results: At the baseline visits, anomalies were detected by multimodal diagnostic tests on all patients. Subjects were followed up for a median duration of 11.5 [3.0–18.7] months. Structural changes at the baseline were detected in 14 of 16 (87.5%) eyes on WAFP and WAFAF and 13 of 16 (81.2%) eyes on SD-OCT. Eight of the ten (80%) eyes that underwent AOSLO imaging depicted structural changes. Functional changes were detected in 14 of 16 (87.5%) eyes on GVF, 15 of 16 (93.7%) eyes on MP, and 11 of 16 (68.7%) eyes on full-field electroretinogram (ff-ERG). Multifocal electroretinogram (mf-ERG) and visual evoked potential (VEP) tests were performed in 14 eyes, of which 12 (85.7%) and 14 (100%) of the eyes demonstrated functional abnormalities, respectively, at baseline. Compared to all the other structural diagnostic tools, AOSLO had a better ability to demonstrate deterioration in retinal microstructures occurring at follow-ups. Functional deterioration at follow-up was detected on GVF in 8 of 10 (80%) eyes, mf-ERG in 4 of 8 (50%) eyes, and MP in 7 of 16 (43.7%) eyes. The ff-ERG and VEP were stable in the majority of cases at follow-up. Conclusions: The utilization of multimodal imaging/tests in the diagnosing and monitoring of npAIR patients can aid in identifying anomalous changes over time. Analysis of both the anatomical and functional aspects by these devices can be supportive of detecting the changes early in such patients. AOSLO shows promise as it enables the capture of high-resolution images demonstrating quantifiable changes to retinal microstructure.

## 1. Introduction

Autoimmune retinopathy (AIR) is a rare immunologic retinal disease characterized by autoantibodies that target antigens found in the retina and/or optic nerve [1]. It is categorized into two groups: paraneoplastic, which is further subdivided into cancer-associated retinopathy (CAR) and melanoma-associated retinopathy (MAR), and non-paraneoplastic. Non-paraneoplastic autoimmune retinopathy (npAIR) has a female preponderance and usually presents with nonspecific vision complaints, such as scotomas, photopsia, subacute vision loss, and nyctalopia, with often unremarkable findings on clinical exam. Few epidemiological studies have suggested that it accounts for about one percent of patients in uveitis/retina clinics [1].

A healthy eye is considered a site of immune privilege, exhibiting reduced immune responses mediated by a multitude of natural defenses, including the blood-ocular barrier (BBB), anterior chamber-associated immune deviation (ACAID), and downregulatory immune environments (DIE) [2]. However, under certain circumstances, this immune privilege can be compromised. Exposure to previously un-exposed antigens or neo-antigens can elicit an immune response, resulting in inflammation. Such a response may worsen subsequent waves of immune reaction and lead to the production of autoantibodies (AAB) [3].

Anti-recoverin, anti-α enolase, and anti-carbonic anhydrase II are examples of AABs that are highly associated with AIR. Studies have established the relationship between these AABs and retinal damage, which occurs via different mechanisms that cause the dysregulation of vital functions responsible for retinal microenvironment homeostasis [4]. These AABs, however, may also be present in various uveitic diseases, cataracts, and normal individuals without ocular diseases. Therefore, it remains controversial to diagnose AIR based solely on AAB positivity [5,6,7,8,9].

Visual field (VF) testing and electroretinography (ERG) can assist in the diagnosis of AIR. VF may show constriction and central or paracentral scotomas, and ERG may appear generically abnormal; however, it lacks specific characteristic findings [1].

Several studies have attempted using optical coherence tomography (OCT) and fundus autofluorescence (FAF) to identify specific patterns or monitor the progression of the AIR [10,11,12]. While these modalities may help in monitoring disease progression, they have not been able to identify disease-specific patterns for AIR. Developments of newer imaging technologies, such as adaptive optics (AO), can provide a better understanding of retinal structural changes.

AO and its newer variant, adaptive optics scanning laser ophthalmoscope (AOSLO), involve correcting light aberrations to increase imaging resolution, enabling the acquisition of high-resolution images that can be used to visualize microstructures such as photoreceptors, microvasculature, nerves, and even the cone-photoreceptor mosaic.

Herein, our study examined eyes diagnosed with npAIR using wide-field imaging techniques, including wide-angle fluorescence angiography (FA), wide-angle FAF, spectral-domain OCT (SD-OCT), AOSLO, Goldmann perimetry (GP), microperimetry (MP), and ERG. By analyzing structural and functional changes over time, we aimed to evaluate each diagnostic modality on its capability to detect the status, course, and prognosis of AIR.

## 2. Materials and Methods

Data from patients diagnosed with npAIR at the Uveitis/Retina clinics at the Byers Eye Institute, Stanford University, were collected from 2017 to 2019. npAIR lacks standardized diagnostic criteria as of the date of this scientific report, and hence, the diagnosis of npAIR is based on the consensus on the diagnosis and management of non-paraneoplastic autoimmune retinopathy using a modified Delphi approach, including a set of four essential criteria (no evidence of malignancy after a thorough work-up, no evidence of degenerative eye disease, a positive screen for anti-retinal autoantibodies, and an ERG abnormality with or without visual field (VF) abnormalities) and five symptoms serving as supportive criteria (photopsia, scotomas, nyctalopia/photo aversion, dyschromatopsia) [13]. The majority (6 of 8) of our patients were enrolled according to these criteria. The remaining two patients were diagnosed based on a high index of clinical suspicion as they matched all the criteria but were negative for anti-retinal antibodies. 

History of photophobia, nyctalopia, dyschromatopsia, or scotomas, with often unrevealing fundus examination with minimal to no intraocular inflammation, abnormal ERGs, OCT, or MP findings, and changes in visual fields can also be supportive of npAIR diagnosis after ruling out other potential differential diagnoses. All patients underwent rigorous laboratory tests to rule out possible etiologies for visual symptoms in a featureless fundus, including CAR and MAR. All subjects had their blood samples’ ARAs analyzed by Immunoblot, immunohistochemistry, and Western blot at the Ocular Immunology Laboratory (Oregon Health and Science University, Portland, OR, USA) [14]. Although anti-retinal antibodies (ARA) were included as a part of the investigation, according to the results of the previously published studies, ARA positivity was considered supportive but not essential for the diagnosis [12,15,16,17,18,19,20,21]. Therefore, we did not consider it mandatory for the enrollment of the patients in our study (2 of 8 patients were ARA negative). 

Patients with a history of cancer, chronic infections, associated autoimmune diseases, and a known history of retinal dystrophy or optic nerve pathology were excluded. This study followed the tenets of the Declaration of Helsinki and was approved by the Stanford University research ethics committee (IRB-41266).

Each patient was imaged utilizing multiple modalities at baseline and follow-ups. Wide-angle fundus photography (WAFP) (Optos Panoramic 200MA™, Optos PLC, Dunfermline, Scotland, UK), Wide-angle fundus autofluorescence (WAFAF) (Optos Panoramic 200MA™, Optos PLC, Dunfermline, Scotland, UK), SD-OCT (Spectralis^®^ (Heidelberg Engineering Inc., Heidelberg, Germany) and AO imaging were done to evaluate the anatomical components and Goldmann perimetry, microperimetry (MAIA (CenterVue Inc., Padova, Italy)), ERG (Diagnosys LLC, Lowell, MA, USA) and visual evoked potential (VEP) (Diagnosys LLC, Lowell, MA, USA) were performed to assess the functional components of the retina.

For AO, we used AOSLO, as described by Dubra and Sulai [22]. The device enables the capture of high-resolution images of the retinal structures. In our study, we imaged photoreceptors. AOSLO images were taken as a video with 150 frames per shot. These were then processed into a single high-resolution image using a semi-automated MATLAB algorithm. Once processed, the images were manually combined using Photoshop software (version 21) in order to create an image montage that allowed for visualization of approximating a 2–3-degree box around the area of central fixation. Image montage was then overlaid onto the fundus infrared (IR) image, and vascular shadows were matched to retinal vessels in order to determine the exact location of the images. A selected point approximately 1 degree from the foveal center was sampled and used for semi-automated cone counting via a custom MATLAB algorithm employing Voronoi tiling, as described in a previous study [23]. To ensure the accuracy of measured changes, sequential images were overlaid and matched [23].

## 3. Results

### 3.1. Patient Demographics

A total of eight patients (16 eyes) met the inclusion criteria and were enrolled. The median age was 46 years (range 7–80), and there was no gender predilection. Seventy-five percent of patients had bilateral symptoms, but severity differed between eyes. The follow-up period varied from 3 to 21 months, with a median of 11.5 months. The demographic details of the patients are depicted in Table 1.

### 3.2. Clinical Course

The most common clinical feature at the initial visit was nonspecific vision problems. Photopsia, reading difficulty (despite presbyopia correction), and reduced field of vision were the most common symptoms (8 of 8 patients). 

Clinically or on fundus examination, the most common cause for suboptimal central vision in eyes with npAIR was macular edema (2 of 16 eyes, 12.5%), followed by foveolar scarring (1 of 16 eyes, 6.25%). On SD-OCT imaging, the most common cause was retinal pigment epithelium (RPE) atrophy, followed by cystoid macular edema (CME). In one patient, CME was a result of Irvine Gass syndrome and was not associated with sub-optimal vision. The other two had transient macular edema, which resolved after topical non-steroidal anti-inflammatory drugs (NSAIDs). 

In three of the six patients with bilateral symptoms, the severity in one eye was found to be different when compared with the contralateral eye. The finding could be suggestive that the npAIR may impact eyes asymmetrically. 

At the end of the follow-up visits, three patients had a gradual worsening of their central vision compared to baseline but maintained a BCVA of equal or better than 20/40 in at least one of their eyes, except for one patient who had a rapid deterioration with the worsening of BCVA from 20/20 to 20/50 within 3 months (Patient 4, OD). Nonspecific visual symptoms persisted among all other patients throughout the follow-up.

### 3.3. Anatomical/Structural Findings via Conventional Imaging in the Cohort

**Figure 1:**
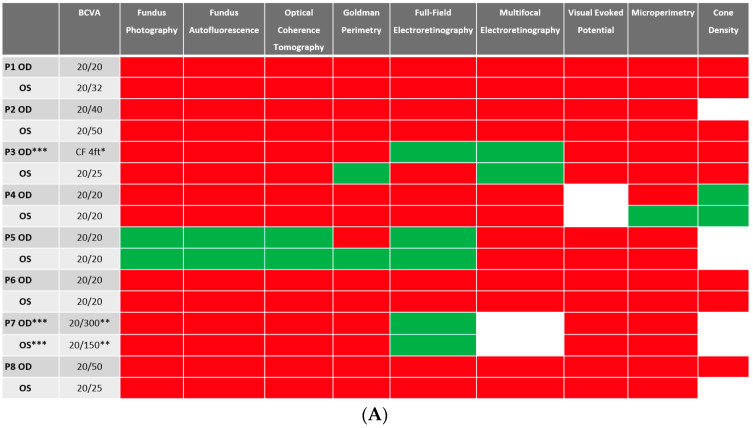

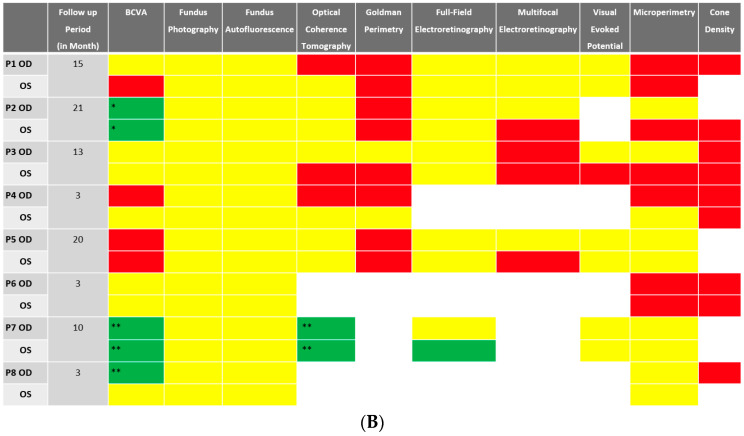
(**A**) Stoplight chart of results from initial imaging. This table illustrates findings seen on baseline imaging in patients whose charts were reviewed. Cells colored in green represent normal results. Cells colored in red represent the presence of abnormal findings. Cells were left blank in cases where the imaging modality was not available or performed. * Poor BCVA is secondary to sub foveal scarring. ** Poor BCVA is secondary to cystoid macular edema. *** As we retrospectively enrolled the patients, all recruited eyes had proof of ERG abnormality at a particular time point; however, those abnormalities were not observed on the initial visit for the selected three eyes. (**B**) Stoplight chart of changes seen on follow-up imaging assessments. This table illustrates changes seen on follow-up imaging in patients whose charts were reviewed. Cells colored in green represent a documented improvement over the follow-up period. Cells colored in yellow represent stable findings with no significant changes over the follow-up period. Cells colored in red represent documented worsening over the follow-up period. Cells were left blank in cases where follow-up imaging was not available. This figure reveals that few imaging modalities can reliably demonstrate changes seen even in extended follow-up periods, emphasizing the importance of utilizing multi-modal imaging in the follow-up of npAIR patients. * Improvement was secondary to cataract surgery. ** Improvement was secondary to the resolution of cystoid macular edema.

Despite BCVA of 20/40 or better in the majority (11 out of 16 eyes) of eyes at baseline, abnormalities were identified in all studied eyes using different imaging modalities. Serial images were obtained at follow-ups, and changes were identified and recorded. A stoplight chart depicting the baseline findings and subsequent improvement, worsening, or stagnation on follow-up are shown in Figure 1A,B, and it reveals how the use of multimodal imaging can detect abnormal findings despite relatively normal best-corrected visual acuity (BCVA) at presentation.

**Figure 2:**
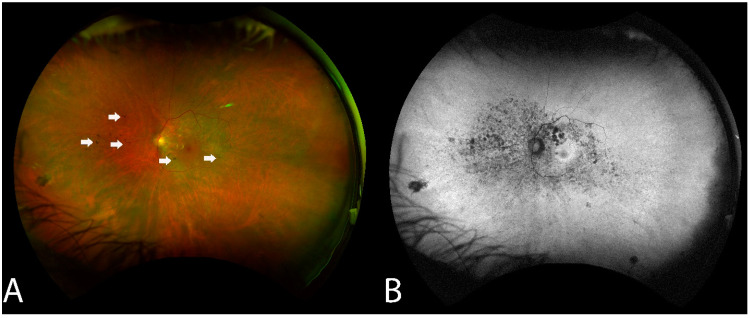
Fundus findings of patient 6 with non-paraneoplastic autoimmune retinopathy (npAIR) at baseline. Color fundus photography illustrates a fundus with a small number of hyperpigmented spicule-like lesions (arrows) scattered around the optic nerve (**A**). Fundus autofluorescence highlights the extent of RPE damage (**B**).

RPE changes mimicking bony spicule-like deposits overlying an area of the hypopigmented retina were observed in 14 of the 16 (87.5%) who underwent WAFP (Figure 2). These changes presented in two general patterns: an incomplete/complete ring around the equatorial retina sparing the central macula or an incomplete/complete ring around the fovea. Interestingly, the extent of lesions did not appear to correlate with BCVA. Compared to WAFP, WAFAF was better able and more informative to highlight the extent of these lesions (Figure 2). However, neither WAFP nor WAFAF depicted any visible changes in any of the patients during their respective follow-up visits, regardless of the status of the progression.

One patient had a lesion involving the foveal center with the presence of foveolar scar (Patient 3). This patient depicted retinal involvement despite being negative for anti-retinal and only being positive for anti-optic nerve autoantibodies to 28-kDa protein.

### 3.4. Spectral-Domain Optical Coherence Tomography

**Figure 3:**
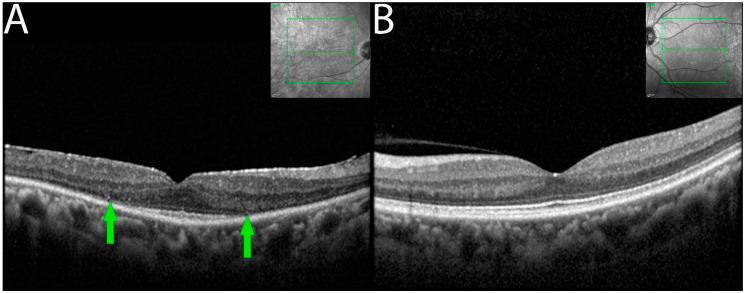
OCT demonstrating ELM and EZ loss in non-paraneoplastic autoimmune retinopathy (npAIR). OCT scans illustrate the right (**A**) and left (**B**) eyes of patient 4 who presented with 20/20 vision in both eyes. The right eye shows diffuse loss of external limiting membrane and ellipsoid zone with the sparing of a central foveal island (area between green arrows). Three months later, the patient’s visual acuity decreased to 20/50.

OCT imaging demonstrated focal or diffuse loss of outer retina involving the external limiting membrane (ELM) and the ellipsoid zone (EZ) in the eyes of seven of the eight patients (Figure 3). Outer retinal involvement was asymmetrical (3 of 6 patients) when occurring bilaterally. Eyes with RPE changes with ELM and EZ losses were associated with larger decreased BCVA compared to eyes having RPE changes alone.

**Figure 4:**
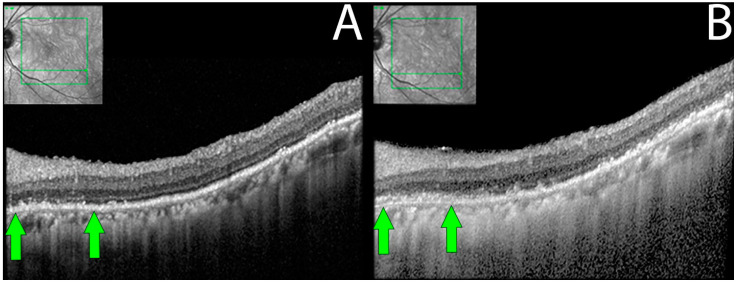
Retinal pigment epithelial changes in non-paraneoplastic autoimmune retinopathy (npAIR). Sequential optical coherence tomography (OCT) imaging in an npAIR patient 5. Column (**A**) shows imaging from the initial visit. Column (**B**) shows imaging on the last documented consult. RPE changes that can be seen include disorganization of normally uniform RPE layer with the layer appearing granulated, migration of RPE to inner retinal structures, areas of focal RPE loss, and increased visibility of Bruch’s membrane signal secondary to RPE losses. Changes can be observed longitudinally (green arrows).

In 9 out of 16 eyes, RPE was disorganized, demonstrating focal losses with sharp delineation over the underlying Bruch’s membrane. In three eyes (18.7%), progressive worsening of RPE/Bruch’s membrane disorganization was also observed during the follow-up period (Figure 4).

### 3.5. Functional Findings of the Cohort

**Figure 5:**
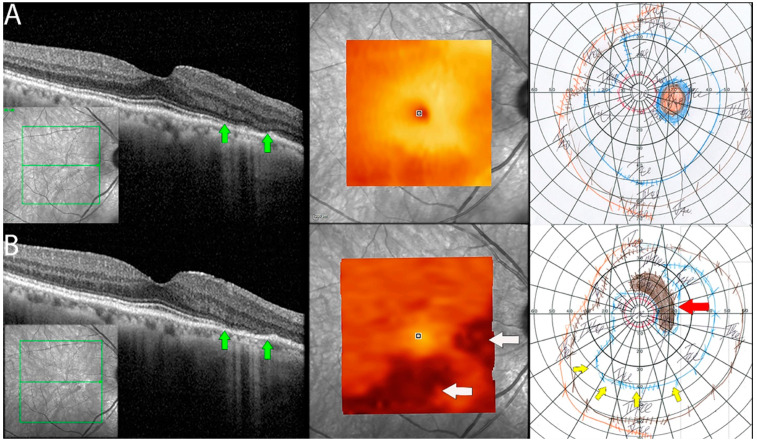
Documented worsening in patient 1 with non-paraneoplastic autoimmune retinopathy (npAIR). Row (**A**) shows right eye results of OCT central B scan, OCT en face scan showing segmented outer retinal layers, and Goldmann perimetry (GP) performed on initial consultation with row (**B**) showing changes 15 months later. The patient had documented worsening. Central OCT scan shows thinning and focal loss of ELM/EZ complex (space between green arrows). This is supported by a segmented en face scan of the outer retina, which shows generalized thinning of the outer retina with the development of focal thinning nasally, with the inferior retina also displaying significant changes compared to the initial scan (white arrows). Follow-up GP shows changes that correlate to OCT findings with the development of blind spot enlargement (red arrow) and constriction of the visual field (yellow arrows).

Goldmann perimetry of npAIR patients mapped various nonspecific findings, which ranged from blind spot enlargement, to focal field defects, to constriction of the visual field. Findings were often asymmetric, correlating to the asymmetrical anatomical involvement as depicted by the test result. Severe constriction of the central and peripheral visual fields to 10–15 degrees was noted in five eyes (31.2%) of three patients. A decrease in central retinal sensitivity was noted to occur in eight eyes (50%) of five patients. Blindspot enlargement (BSE) was either seen initially or noted to develop in six eyes (37.5%) of five patients, in which three of the six eyes had further BSE on follow-up. GP findings were also found to be in correlation with the subjective complaints and worsening anatomical parameters detected via structural imaging modalities, and one such representative case is illustrated in Figure 5.

Electrophysiologic testing revealed decreased amplitudes on ff-ERG test results during the initial consultation in six out of the eight (66.6%) patients. Cone and rob b-wave amplitudes were found to be decreased. Rod b-wave amplitudes were often found to be worse than cone amplitudes. Totally extinguished rod amplitudes were seen in nine eyes (56.3%), and totally extinguished responses in both cone and rod amplitudes were seen in four eyes (25%). In our study, BCVA did not correlate with ff-ERG values when 20/32 or better. ff-ERG findings were stable, with mild fluctuations in most patients throughout the follow-up period.

Worsening of mf-ERG on follow-up was noted when peripheral rings were significantly involved. The finding also correlated similarly in four other eyes and were in coherence with subjective complaints of worsening or with detectable changes in diagnostic modalities.

Asymmetric VEP abnormalities were initially noted in both eyes of all patients initially imaged (seven of seven). VEP demonstrated low amplitudes in eleven (78.6%) eyes of seven patients, blunted waveforms in five eyes (35.7%) of three patients, and delays in latency ranging from mild to markedly abnormal were seen in eleven (78.6%) eyes of six patients. On review of the follow-up results, the majority (87.5%) of VEP findings were noted to be stable (4 of 5 patients).

**Figure 6:**
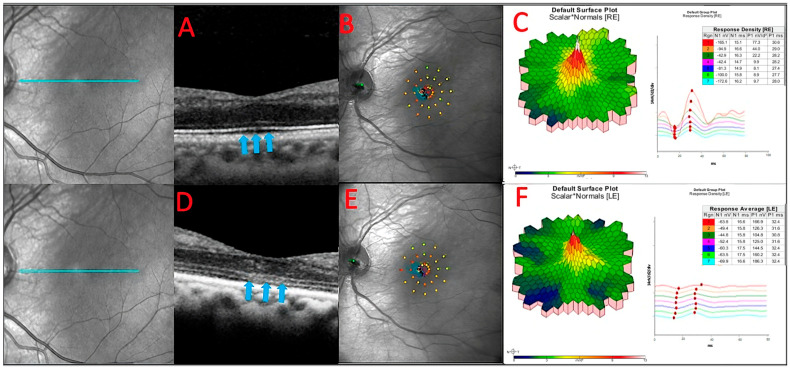
The baseline OCT of patient 3 (**A**). He had a slow sub foveal development of new focal ELM and EZ losses as well as a slow extension of existing lesions over a 13-month period, despite receiving therapy (blue arrows) (**D**). These structural changes corresponded to physiological changes detected by microperimetry (**B**,**E**) and multifocal ERG (**C**,**F**), which depicted an overall decrease in the retinal sensitivity to visual stimulus.

MP revealed severely reduced central retinal sensitivity in the majority (87.5%) of patients at the initial visit. Nearly half (7 of 16 eyes) had a generalized reduction in retinal sensitivity, affecting parafoveal more than central fields. Seven eyes (87.5%) of four patients had worsening micro-scotomas, which also correlated with findings such as ELM/EZ loss on OCT and/or subjective worsening (Figure 6).

### 3.6. Adaptive Optics Scanning Laser Ophthalmoscopy

The use of AOSLO for cone quantification was attempted in six (75%) patients. Images suitable for cone quantification were obtained from ten eyes (83.3%) at the initial and follow-up visits. Quantification from the other eyes could not be established due to the low quality of the acquired images. Results are illustrated in Table 2.

**Figure 7:**
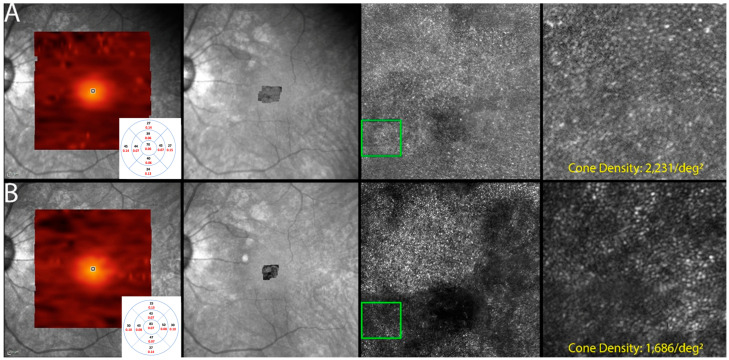
Optics scanning laser ophthalmoscopy (AOSLO) and detectable OCT changes in non-paraneoplastic autoimmune retinopathy (npAIR); images are from patient 2. This figure illustrates follow-up changes in the left eye of an npAIR patient (patient 2). Row (**A**) shows imaging performed at baseline, and row (**B**) shows follow-up imaging conducted at month 15. En face OCT showed a segmented outer retina with sparing of the central island and generally reduced outer retina thickness indices in the follow-up visit. The use of AOSLO enables visualization of the cone-photoreceptor mosaic. Semi-automated cone quantification of the preselected area in this cone-photoreceptor mosaic showed a decrease in cone density, which corresponds with retinal OCT findings.

Cone density was found to be decreased in all ten eyes that underwent imaging. Eyes with grossly visible changes in the photoreceptor mosaic image were in coherence with the deteriorating status of the macula, which was depicted by imaging modalities such as SD-OCT, showing EZ loss and also with subjective worsening (Figure 7).

## 4. Discussion

Non-paraneoplastic autoimmune retinopathy (npAIR) is a complex retinal disease to diagnose and manage, as most cases present with normal-appearing eyes, nonspecific visual symptoms, and no signs of active inflammation [24].

Detection of circulating autoantibodies against retinal and/or optic nerve antigens can help in the diagnosis of npAIR, although it is not an essential criterion for diagnosis [24,25]. Such assessment is mainly attributed to the heterogeneity of autoantibodies present in tested npAIR patients [21]. Additionally, due to the lack of a universally standardized antiretinal antibody testing assay, there are often inconsistencies in results and subsequent diagnoses among laboratories and clinicians [15]. Therefore, it may be prudent to employ a single laboratory for testing in practice to ensure achieving consistent results. Additionally, screening these patients meticulously for malignancy to rule out other genetic, infectious, and immune-mediated disorders along with CAR and MAR is essential.

Recent advances in imaging and diagnostic techniques, such as FP, FAF, FA, OCT, ERG, and MP, have facilitated the identification of characteristic changes relating to the gross loss of photoreceptors affecting the retinal function. However, it is still difficult to designate these findings as “hallmarks” of npAIR due to variations in results [10,26,27]. Recently, a consensus was made by the American Uveitis Society (AUS) that the use of FAF, FA, OCT, and ERG can be helpful in diagnosing AIR [13]. The AUS also highlighted that the correlation of findings from multiple imaging modalities is more supportive of a diagnosis [13].

All of the patients in our study had nonspecific visual symptoms at presentation, as previously described in the literature [1,28]. Photopsia and difficulty in reading were present in all patients, and our findings were consistent with what has been described in the literature [6,24,29]. Cystoid macular edema was the most common cause of suboptimal BCVA in npAIR patients. Such a finding is also consistent with a previous report from Ferreyra et al. in which 45.8% of the included patients with npAIR and suboptimal BCVA had CME [30,31]. Another study by Finn and colleagues not only further supported these findings, but also suggested that the suboptimal vision could be the result of the EZ loss in the foveal region as a result of the CME [31].

Several studies have shown abnormal findings in WAFP images of AIR patients, as in our study [32]. However, it is extremely challenging to diagnose npAIR using these findings due to them being nonspecific. Most previously described findings have been limited to RPE clumps or the presence of “bony spicule”-like deposits. WAFAF revealed lesion margins better compared to WAFP. Although WAFAF has been a great imaging technique in evaluating the progression of other retinal disorders, it was not helpful in delineating the progression of disease in our study. The images from WAFP and WAFAF appeared stable throughout follow-up periods in all our patients, despite structural changes in SD-OCT and functional changes in VF, MP, and ERG.

A study by Khanna et al. reported that 22% of their npAIR patients’ eyes had normal funduscopic appearances at the initial visit as assessed by FP. Similarly, on evaluation by FAF, only 20% of the eyes had normal FAF appearances. However, the study did not mention the use of these assessments to monitor and track disease progression at follow-ups [32].

SD-OCT is useful in analyzing anatomical changes during follow-up evaluations, and the findings from our study were no different. Gradual loss of the ELM/EZ complex followed by RPE disorganization and progressive focal losses were visualized on SD-OCT. Additionally, these findings were coherent with the associated functional/ physiological changes. In severe cases, patients were left with a central island of intact ELM/EZ, which most often corresponded to constricted visual fields. Similar findings have been documented by Lima et al. and Khanna et al., who also concluded that the loss of ELM/EZ complex with RPE disorganizations is suggestive of npAIR disease progression [10,32].

However, due to the thin nature of layers involved and still many uncertainties looming on the exact location where AIR affects the retina, changes seen longitudinally may not be reliably quantifiable as they may be minimal (less than 10-micron differences) with no overall changes in retinal volume in the majority of cases. With the introduction of high-resolution and next-generation OCTs, these changes may be better detected, which may also help us to understand the “micro pathologies” better.

Our study also revealed that while RPE changes may be seen in cases with diffuse EZ/ELM loss, the RPE changes alone do not seem to significantly impact BCVA. Identifying early RPE changes (when occurring alone) highlights the importance of identifying “pre-symptomatic” signs to detect the disease before it progresses and causes further damage, as visual acuity may not be affected early in the disease course. Use of devices that measure functional components of the retina might be helpful in this regard.

Goldmann perimetry (GP)/visual field, in particular, is effective at documenting disease progression across patients during follow-up evaluations. Gradual visual field constriction, blind spot enlargement, and decreased central retinal sensitivity were noted in almost all the eyes of our patients at follow-up visits. Of the modalities used, GP appears to be the most capable of detecting changes on follow-up. The AUS has acknowledged the possible utility of GP in npAIR but did not reach a consensus on its use as an essential modality for measuring disease progression [13]. Although MP is a subjective test, from the congruity of the findings elicited by the patients in our longitudinal study, it can be suggested that GP can elucidate subtle changes related to disease progression during follow-up evaluations.

MP is capable of measuring retinal sensitivity using the fundus-correlated perimetric technique, and is particularly used to monitor macula-involving retinal diseases such as age-related macular degeneration [33,34,35]. It is also capable of measuring instabilities in central fixation [36,37]. In our study, we used MP to monitor functional changes in the macula, and GP to measure functional changes in the peripheral visual field. There are few studies that have utilized MP to monitor patients with AIR. Most of these studies have adopted MP to study retinal function in paraneoplastic AIR [10,38,39]. In our study, MP was able to provide consistent and reliable information regarding losses in retinal sensitivity among eyes with progressive npAIR.

Our study elicited that MP has a temporal advantage over other modalities as it can detect a decrease in retinal sensitivity before a decrease in visual acuity occurs. In one patient with mild disease, BCVA remained at 20/32 or better in both eyes throughout the follow-up period of 20 months, despite the subjective vision worsening described by the patient. However, the use of GP and mf-ERG documented a slow but continuous deterioration of peripheral vision in both eyes. MP is one device that has the potential to be used in assessing the progression of npAIR. MP changes are in agreement with anatomical changes observed in SD-OCT scans of the same eyes.

ff-ERG is a sensitive tool for detecting retinal pathology that demonstrates specific changes that aid in distinguishing CAR, MAR, and npAIR [40]. In our study, three patients had extinguished or near extinguished ff-ERG results at baseline, which made it difficult to elicit further progression. In the eyes of other patients, the concurrent use of mf-ERG appeared to be capable of showing the changes and was able to reveal residual central and low peripheral on follow-up. However, similar results should be interpreted cautiously, as even in the presence of extinguished rod and cone responses on ff-ERG and mf-ERG, one of the patients had a BCVA of 20/32 or better in both eyes throughout the follow-up. Similar findings have been noted in patients with retinitis pigmentosa, suggesting that residual electrical retinal activity may exist in these cases that are undetectable by the currently available ERG machines [40]. Such a finding emphasizes that ERG alone might have limitations, underscoring the need for multiple functional modalities to confirm the diagnosis or progression of npAIR.

In contrast to the utilization of ERG in npAIR, there is a paucity in the literature regarding the use of VEP for the diagnosis and follow-up of npAIR patients [41,42,43]. The VEP results in all of our patients showed abnormality at the baseline visit, which is similar to a report from Fox et al [42]. However, VEP had limitations in the follow-up visit, as it could not detect the changes in the majority (7 of 8) of the eyes, despite other modalities depicting the progression of npAIR,

Once an npAIR diagnosis has been established, a challenge lies in determining how best to monitor these patients. Though our study demonstrated that many imaging modalities reveal abnormalities at the initial screening visit of npAIR patients, no single modality demonstrated significant changes throughout follow-up periods.

Interestingly, BCVA remained remarkably consistent in our patient population, despite the progression of pathology on functional imaging. Such observation is likely a result of central island sparing as visualized on OCT. Other studies have documented similar OCT changes in chronic npAIR patients [21,26,27,28,44]. In three (37.5%) of our cases with severe npAIR, and with a follow-up period of more than one year, the use of multi-modal imaging documented worsening anatomical and functional components, despite little to no subjective changes reported by the patients. In general, functional tests such as MP appear to be more sensitive in their ability to detect changes longitudinally than structural modalities in npAIR patients, as determined in our study.

AOSLO is a novel imaging technique that captures high-resolution images, allowing for the visualization of retinal microstructures directly. Our study is one of the few that has explored whether AOSLO can be used as a diagnostic/trend-analyzing tool in npAIR. Analysis of the cone-photoreceptor mosaic in standard size approximately 1 degree from the fovea in cases of npAIR revealed abnormal cone–photoreceptor morphology and decreased density on quantification. The data are similar to previous findings of another study that had imaged AIR patients using a commercially available AOSLO device [44]. In a case report, Williams et al. demonstrated a high correlation and accuracy between in vivo AOSLO results and the retina histology specimen finding of a CAR patient when the density of cone cells was evaluated [45].

Our study suggests that AOSLO has the potential to monitor disease progression in severe npAIR patients, as it enables quantification of retinal structures such as photoreceptors. Our study also demonstrates that cone density decreased as npAIR progressed. However, as our study population is small, further studies with larger sample sizes and more extended follow-up periods should be explored to validate our findings and explore what AOSLO may have in monitoring patients with npAIR.

The limitation of our study is that it is a single-center, retrospective study with patients having follow-up visits with a wide range of duration. Although various studies have suggested that ARAs titers are not necessarily positive in npAIR patients due to their low sensitivity and specificity, they are still considered one of the primary supportive pillars of the diagnosis, which were not positive in two of our patients.

## 5. Conclusions

The management of patients with npAIR is challenging, often complicated by delayed diagnosis due to a multitude of factors. Our longitudinal study and review of literature magnify the importance of high clinical suspicion and the use of multi-modal imaging in order to diagnose, prognosticate, and optimize visual outcomes in such patients.

When used in appropriate combination, these diagnostic imaging devices will often show anomalies, which can aid in increased clinical suspicion and diagnosis. Fundus photography and fundus autofluorescence are more capable of highlighting subtle changes that may be missed in clinical examination. The use of SD-OCT further characterizes these changes by revealing demonstrable characteristic changes in the outer retinal layers with diffuse losses of the ELM/EZ complex and sparing of the central island in severe disease. However, structural modalities are often unable to reliably demonstrate significant changes over time.

The findings from these devices can be further coupled with findings from functional modalities, particularly GP and ERG (full-field and multifocal), for better understanding and clinical decisions. MP is a very reliable tool, but it is important to note that npAIR patients often display losses in the peripheral fields and enlarged blind spots, suggesting peripheral retinal involvement initially and later progressing to the central retina.

Ultimately, AOSLO is an emerging modality that shows promise in monitoring severe cases of npAIR. AOSLO, in particular, may prove to be important as it appears to be able to demonstrate and possibly quantify microstructural changes that other conventional structural imaging modalities cannot replicate. AOSLO can be one of the reliable imaging modalities that may be considered in the management of npAIR patients.

## Figures and Tables

**Table 1:** Demographics of the patients with diagnosis of non-paraneoplastic autoimmune retinopathy (npAIR).

	Age	Sex	Race/ Ethnicity	Chief Complaint	(+) Antibodies	Total Months Followed Up
1	66	F	Asian/ Non-Hispanic	Nonspecific blurring of vision OU	Retina: Recoverin, Rab6, Aldolase, Enolase, HSP60	15
					ON: None noted	
2	67	F	Caucasian/Non-Hispanic	Nonspecific blurring of vision OU	Retina: GAPDH, Aldolase, Enolase, 58-,68-, 98-kDa	21
					ON: None noted	
3	13	M	Caucasian/Non-Hispanic	Rapidly deteriorating vision OD	Retina: Negative on testing	13
					ON: 28-kDa	
4	59	M	Asian/ Non-Hispanic	Nonspecific blurring of vision OD	Retina: Negative on testing	3
					ON: 30-, 36-, 41-, 44-kDa	
5	23	M	Caucasian/Non-Hispanic	Nonspecific field deficits OU Transient scotomas OD	Retina: GAPDH	20
					ON: 35-, 136-kDa	
6	33	M	Asian/ Hispanic	Visual field constriction OU Photopsias OU	Retina: 35-, 46-, 62-kDa	3
					ON: 35-, 46-, 62-kDa	
7	7	F	Caucasian/ Non-Hispanic	Nonspecific blurring of vision OU	Retina: Enolase	10
					ON: 22-kDa	
8	80	F	Caucasian/ Hispanic	Visual field constriction OU Previous diagnosis of AIR	Retina: Enolase	3
					ON: None noted	

**Table 2:** Mean cone densities of patients with non-paraneoplastic autoimmune retinopathy (npAIR).

Patient (Eye)	Duration of Follow-Up (in Months)	Mean Cone Density (Cones per deg^2^)	
		Initial Visit	Final Visit
1 (Right eye)	9	2317	1981
1 (Left eye)	0 *	1884	NA *
2 (Left eye)	8	2231	1686
3 (Right eye)	13	1988	1295
3 (Left eye)	13	2453	2048
4 (Right eye)	2	7141	5561
4 (Left eye)	2	6715	5952
6 (Right eye)	3	2226	1978
6 (Left eye)	3	2123	1871
8 (Right eye)	2	2046	1838

* There was no available AOSLO image of patient’s left eye with gradable quality on follow up visit.

## Data Availability

Data will be available to readers who request via email.
